# Association of Pretreatment Anemia with Pathological Response and Survival of Breast Cancer Patients Treated with Neoadjuvant Chemotherapy: A Population-Based Study

**DOI:** 10.1371/journal.pone.0136268

**Published:** 2015-08-20

**Authors:** Wenjie Zhu, Binghe Xu

**Affiliations:** Department of Medical Oncology, Cancer Hospital, Chinese Academy of Medical Sciences and Peking Union Medical College, Beijing, P. R. China; University of Florida, UNITED STATES

## Abstract

**Background:**

Anemia related to adjuvant chemotherapy might predict compromised survival in patients with breast cancer. The present population-based study was to investigate the correlation of pretreatment anemia with pathological response and long-term prognosis of breast cancer patients receiving neoadjuvant chemotherapy (NCT).

**Methods:**

From 1999 to 2011, a total of 655 patients with operable or locally advanced breast cancer who underwent NCT before definitive surgery were reviewed. The patients were subdivided into anemic (baseline hemoglobin (Hb)<12.0g/dL) and non-anemic (Hb≥12.0g/dL) groups. Comparison was made between anemic and non-anemic groups concerning the rate of pathological complete response (pCR), relapse-free survival (RFS), overall survival (OS) and cancer-specific survival (CSS). Logistic and Cox regression models were utilized to determine the predictive value of pretreatment anemia in outcomes of patients undergoing NCT.

**Results:**

166 women (25.3%) were anemic before treatment. Patients in the anemic group were less likely to achieve pCR in NCT than their non-anemic counterparts (odds ratio (OR) 0.428, 95% confidence interval (CI) 0.198–0.927, p = 0.031). Patients with baseline anemia displayed inferior 10-year RFS (59.1% vs 66.0%, p = 0.022 by log-rank), OS (75.3% vs 90.9%, p<0.001) and CSS (82.4% vs 94.4%, p<0.001) compared with those without. After adjustment for confounders, pretreatment anemia was demonstrated to correlate with elevated risk of relapse (hazard ratio (HR) 1.453, 95% CI 1.077–1.962, p = 0.015), cancer-specific mortality (HR 2.961, 95% CI 1.679–5.222, p<0.001) and all-cause mortality (HR 2.873, 95% CI 1.757–4.699, p<0.001).

**Conclusions:**

Pretreatment anemia was associated with worse pathological response to NCT as well as survival status in breast cancer. Further studies are warranted to identify optimal interventions and improve the prognosis of this subgroup.

## Introduction

Anemia is a syndrome prevalent in patients with cancer. According to earlier reports, an estimated 30–90% of cancer patients are complicated with anemia [1]. Previous studies have identified anemia as an independent prognostic factor, which has an adverse influence on survival in several types of cancer including breast cancer [2,3]. In a population-based study involving 2,690 patients with operable breast cancer, preoperative anemia was associated with worse relapse-free survival (RFS) as well as overall survival (OS) [4]. The compromised survival observed among cancer patients with anemia has been mainly attributed to decreased capacity of oxygen transport and thus tumor hypoxia [5]. It is well established that hypoxia in tumor mass is a major therapeutic obstacle in radiotherapy since cancer cells tend to be resistant to ionizing radiation under hypoxic conditions [6]. Baseline hemoglobin (Hb) levels have been found to correlate with outcomes of radiotherapy in patients with cancer of head and neck [7], cervix [8], prostate [9] and breast [10]. In addition, hypoxic environment induces cancer cells into a quiescent state and decreases the fraction of cellular proliferation, thus rendering tumor less responsive to cytotoxic agents in chemotherapy [11]. Previous findings indicated that anemia might be predictive of worse treatment outcomes of chemotherapy in breast cancer [12]. However, most studies were performed in the adjuvant setting, where a direct evaluation of tumor responsiveness to chemotherapy was lacking.

Neoadjuvant chemotherapy (NCT) administered prior to definitive surgical resection is recommended as the standard of care in locally advanced breast cancer. In recent years, expanded use of NCT has been seen in even early stage breast cancer cases. It shrinks the tumor mass and increases the odds of surgical removal of tumors that are initially inoperable. Besides, NCT presents itself as an optimal model of assessing treatment efficacy in vivo and identifying potential response-related clinical or biological factors which are able to discriminate chemo-resistant cancer cases from sensitive ones. A few studies investigated the association of pretreatment Hb concentrations with response to NCT in breast cancer patients but inconsistent findings were obtained. Bottini et al reported that low Hb levels (≤13g/dL) negatively influenced response to NCT in breast cancer possibly via inhibition of anti-proliferative activity [13]. Another cohort study however, failed to observe any statistically significant association between Hb levels and clinical response to NCT [14]. Whether or not baseline anemia could contribute to poor treatment outcomes in the neoadjuvant setting has yet to be elucidated. It is noteworthy that previous studies were mostly retrospective based on small cohorts, which merits further research in expanded population.

In the current study we evaluated anemia before treatment as a predictive variable for response to NCT and as a prognostic factor in patients with breast cancer. The primary aim of this cohort study was to determine the impact of pretreatment anemia on pathological response to NCT, and the secondary aim was to illuminate the relationship between initial anemia and relapse or survival of these patients.

## Methods

### Study population

From January 1999 to December 2011, a total of 655 consecutive patients with operable or locally advanced breast cancer who received NCT at Cancer Hospital, Chinese Academy of Medical Sciences and Peking Union Medical College (Beijing, China) were enrolled in the present study. Medical records were reviewed and relevant information was retrospectively collected including demographic characteristics, tumor presentation, histopathologic assessment and therapy details. Diagnosis with invasive carcinoma was confirmed by core needle biopsy (CNB) and the lymph node status was evaluated by fine needle aspiration (FNA) of palpable lymph nodes if applicable. Before the initiation of NCT, bilateral breast MRI or ultrasound, chest X-ray, abdominal ultrasound or CT scan and bone scintigraphy were performed to determine clinical staging. Stage IV disease, bilateral breast cancer, male breast cancer, inflammatory breast cancer or patients complicated with other malignancies were excluded from the current analysis. The study was approved by the institutional review board (IRB) of Cancer Hospital, Chinese Academy of Medical Sciences and Peking Union Medical College. Informed consent requirements were waived by the IRB since an honest broker provided a de-identified dataset for the analysis.

The last Hb measurement within 2 weeks before initiation of NCT was set as pretreatment Hb level and incorporated into the present analysis. We defined pretreatment anemia as Hb<12.0g/dL according to the World Health Organization (WHO) criteria, and the patients were then subdivided into anemic (Hb<12.0g/dL) and non-anemic (Hb≥12.0g/dL) groups.

### Treatment, response evaluation and follow-up

All participants underwent 4–6 cycles of NCT with AT (Doxorubicin 50g/m^2^ IV day1, Paclitaxel 175mg/m^2^ IV day2, q21d) or ET (Epirubicin 75g/m^2^ IV day1, Paclitaxel 175mg/m^2^ IV day2, q21d) regimens before surgery. Other preoperative anti-cancer treatments such as endocrine therapy and radiotherapy were precluded in this study. Within one month after completion of NCT mastectomy or breast-conserving surgery was performed in association with sentinel lymph node biopsy (SLNB) or axillary lymph node dissection (ALND). After surgery the patients received another 2–4 cycles of adjuvant chemotherapy to fulfill a total of 6–8 cycles of chemotherapy when indicated. Adjuvant radiotherapy was prescribed at the discretion of physicians according to clinical as well as pathological assessment, followed by endocrine therapy in cases with hormone receptor positive tumors. For patients with human epidermal growth factor receptor-2 (HER-2) over-expressing tumors, Trastuzumab was recommended in adjuvant setting alone or in combination with chemotherapy.

Evaluation of clinical response to NCT was carried out for every two cycles of chemotherapy based on physical and imaging examinations (breast MRI or ultrasound). Pathological response was assessed by two independent pathologists and pathological complete response (pCR) was defined as the absence of invasive carcinoma from both the breast and lymph nodes of the resected specimen. Residual ductal carcinoma in situ (DCIS) was factored into the pCR group.

Follow-up visits at Cancer Hospital were scheduled every 3 months during the first year, every 6 months for the next 2 years and annually thereafter. History and physical examinations were obtained every 6 months for the first 5 years. Mammogram, chest X-ray and gynecologic assessments (if applicable) were performed every 12 months during the first 5 years. Then after 5 years only physical examinations, mammogram and gynecologic assessments (if applicable) were required annually. For patients with regional disease, chest CT, abdominal±pelvic ultrasound and bone scan were evaluated yearly to monitor for metastatic relapse. Data on individual vital status and causes of death was collected by regular telephone surveys and medical charts review. Minimum duration of follow-up was two years.

### Immunohistochemistry and pathology

Pathological evaluation and determination of intrinsic subtypes utilizing tumor specimens from CNB was completed prior to treatment. Immunohistochemistry (IHC) was carried out to decide the status of estrogen receptor (ER), progesterone receptor (PR) and HER-2. ER or PR positivity was defined as at least 1% of tumor cells were positive with nuclear staining. HER-2 expression status was described as 0, 1+, 2+ and 3+ by IHC, and tumors were HER-2 positive if they were reported as 3+ by IHC or positive by florescent in situ hybridization (FISH). Otherwise the tumors were HER-2 negative.

### Statistical analysis

The Chi-squared test was performed to compare the distribution of patient characteristics between anemic and non-anemic groups. A multivariate logistic regression model was used to determine the association between pCR and potential predictive factors. RFS was defined as the interval between initiation of NCT and the date of disease relapse or death from any cause. OS was calculated from the date of treatment initiation to the date of death. Cancer-specific survival (CSS) was determined from the date of NCT initiation to the date of breast cancer-specific death. Cases without relapse or death events were censored at the date of last follow-up. Survival curves were estimated using the Kaplan—Meier method and unadjusted comparison of these estimates was made using log-rank test. Cox proportional hazards regression model was utilized to identify independent prognostic factors. All p values reported were two-sided with p<0.05 being considered statistically significant. All statistical analyses were performed with SPSS version 19.0 (SPSS Company, Chicago, IL).

## Results

### Pretreatment anemia and patient characteristics

In all, 655 women with breast cancer were enrolled in this cohort study. The median age of study population was 48 years old (range 23–78 years). 60.6% of the patients were premenopausal. 166 women (25.3%) were anemic before treatment which corroborated previous report about the prevalence of initial anemia [4]. As showcased in Table 1, no significant difference was observed in the distribution of clinical and histopathologic characteristics between anemic and non-anemic groups. Inter-group comparison was also performed concerning the systemic treatment status. Individuals from either group received a median of 4 cycles of chemotherapy (range 4–6). Instead of standard dosing protocols, decreased therapy dosing was administered in 9.6% of anemic patients and 9.0% of non-anemic ones (p = 0.805) at the first cycle, as a result of low baseline Hb level (<90g/dL) or competing conditions such as cardiovascular diseases. Also, there was no evident disparity in the incidence of toxicity-associated dose reduction or delay during treatment between groups (7.8% vs 8.2%, p = 0.887). Moreover, the rates of postoperative treatment were well balanced between two groups, which consisted of adjuvant chemotherapy (81.9% vs 80.2%, p = 0.619), radiotherapy (74.7% vs 76.7%, p = 0.603), endocrine (67.5% vs 63.6%, p = 0.368) and targeted anti-HER2 therapy (36.2% vs 34.9%, p = 0.245).

**Table 1:** Patient characteristics by pretreatment anemia status.

N (%)	All	Hb<12g/dL	Hb≥12g/dL		
	N = 655 (100%)	N = 166 (25.3%)	N = 489 (74.7%)	χ^2^	p
Age					
≤50 y	397(60.6)	108(65.1)	289(59.1)	1.844	0.174
>50 y	258(39.4)	58(34.9)	200(40.9)		
Menopause					
No	397(60.6)	111(66.9)	286(58.5)	3.646	0.056
Yes	258(39.4)	55(33.1)	203(41.5)		
BMI					
<25	339(51.8)	87(52.4)	252(51.5)	0.038	0.845
≥25	316(48.2)	79(47.6)	237(48.5)		
Histology					
Invasive ductal	592(90.4)	149(89.8)	443(90.6)	0.231	0.891
Other	63(9.6)	17(10.2)	46(9.4)		
Histologic grading					
G1, G2, or G x	442(67.5)	111(66.9)	331(67.7)	1.982	0.371
G3	213(32.5)	55(33.1)	158(32.3)		
Estrogen receptor					
Positive	382(58.9)	98(59.8)	284(58.6)	0.073	0.787
Negative	267(41.1)	66(40.2)	201(41.4)		
Progesterone receptor					
Positive	382(58.9)	95(57.9)	287(59.2)	0.079	0.779
Negative	267(41.1)	69(42.1)	198(40.8)		
HER-2 overexpression					
Positive	227(34.7)	58(34.9)	169(34.6)	0.153	0.926
Negative	376(57.4)	96(57.8)	280(57.3)		
Unknown	52(7.9)	12(7.2)	40(8.2)		
Clinical tumor stage					
T1	26(4.0)	6(3.6)	20(4.1)	2.022	0.568
T2	272(41.5)	65(39.2)	207(42.3)		
T3 and T4	357(54.5)	95(57.2)	262(53.6)		
Clinical node status					
N0	152(23.2)	33(19.9)	119(24.3)	1.381	0.240
≥N1	503(76.8)	133(80.1)	370(75.7)		
Clinical stage					
II	269(41.1)	60(36.1)	209(42.7)	2.228	0.136
III	386(58.9)	106(63.9)	280(57.3)		
Pathologic tumor stage					
T0	91(13.9)	15(9.0)	76(15.5)	5.160	0.271
T1	288(44.0)	74(44.6)	214(43.8)		
T2	194(29.6)	52(31.3)	142(29.0)		
T3 and T4	82(12.5)	25(15.0)	57(11.6)		
Pathologic node status					
N0	216(33.0)	54(32.5)	162(33.1)	0.245	0.970
N1	182(27.8)	48(28.9)	134(27.4)		
N2	137(20.9)	33(19.9)	104(21.3)		
N3	120(18.3)	31(18.7)	89(18.2)		
Pathologic stage					
0	66(10.1)	9(5.4)	57(11.7)	5.685	0.128
I	89(13.6)	26(15.7)	63(12.9)		
II	220(33.6)	58(34.9)	162(33.1)		
III	280(42.7)	73(44.0)	207(42.3)		
Type of surgery					
Mastectomy	593(90.5)	156(94.0)	437(89.4)	3.073	0.080
Breast-conserving surgery	62(9.5)	10(6.0)	52(10.6)		
Lymphovascular invasion					
No	551(84.1)	137(82.5)	414(84.7)	0.422	0.516
Yes	104(15.9)	29(17.5)	75(15.3)		
Adjuvant chemotherapy					
No	127(19.4)	30(18.1)	97(19.8)	0.247	0.619
Yes	528(80.6)	136(81.9)	392(80.2)		
Adjuvant radiotherapy					
No	156(23.8)	42(25.3)	114(23.3)	0.270	0.603
Yes	499(76.2)	124(74.7)	375(76.7)		
Endocrine therapy					
No	232(35.4)	54(32.5)	178(36.4)	0.812	0.368
Yes	423(64.6)	112(67.5)	311(63.6)		
Comorbidity					
No	538(82.1)	143(86.1)	395(80.8)	2.433	0.119
Yes	117(17.9)	23(13.9)	94(19.2)		

### Pretreatment anemia and pathological response to NCT

66 (10.1%) out of 655 patients achieved pCR after NCT. As exhibited in Table 2, in anemic group the rate of pCR was only 5.4%, which was considerably lower than that of non-anemic group (11.7%; p = 0.024 by Chi-squared test). Logistic regression model was applied in order to disclose any association between pCR and potential predictive variables. After adjustment for confounders including age, menopausal status, BMI, histology, grade, ER, PR, HER-2 expression, tumor size, node status, clinical stage and comorbidity, anemia remained indicative of decreased likelihood of pCR (odds ratio (OR) 0.428, 95% confidence interval (CI) 0.198–0.927, p = 0.031). Moreover, age (OR 4.805, 95% CI 1.845–12.515, p = 0.001) and ER status (OR 4.199, 95% CI 1.904–9.261, p<0.001) were revealed to be independent predictive factors of pCR. However, we failed to observe significant association between baseline Hb levels and pathological response to NCT when Hb was evaluated as a continuous variable (OR 1.007, 95% CI 0.990–1.024, p = 0.452).

**Table 2:** Outcomes of patients by pretreatment anemia status.

N (%)	All	Hb<12g/dL	Hb≥12g/dL		
	N = 655 (100%)	N = 166 (25.3%)	N = 489 (74.7%)	χ^2^	p
pCR					
No	589(89.9)	157(94.6)	432(88.3)	5.317	0.021
Yes	66(10.1)	9(5.4)	57(11.7)		
Relapse					
No	452(69.0)	104(62.7)	348(71.2)	4.201	0.040
Yes	203(31.0)	62(37.3)	141(28.8)		
Cancer-specific death					
No	607(92.7)	143(86.1)	464(94.9)	13.949	<0.001
Yes	48(7.3)	23(13.9)	25(5.1)		
Death					
No	591(90.2)	135(81.3)	456(93.3)	19.994	<0.001
Yes	64(9.8)	31(18.7)	33(6.7)		

Abbreviations: pCR: pathological complete response.

### Pretreatment anemia and survival

**Fig 1:**
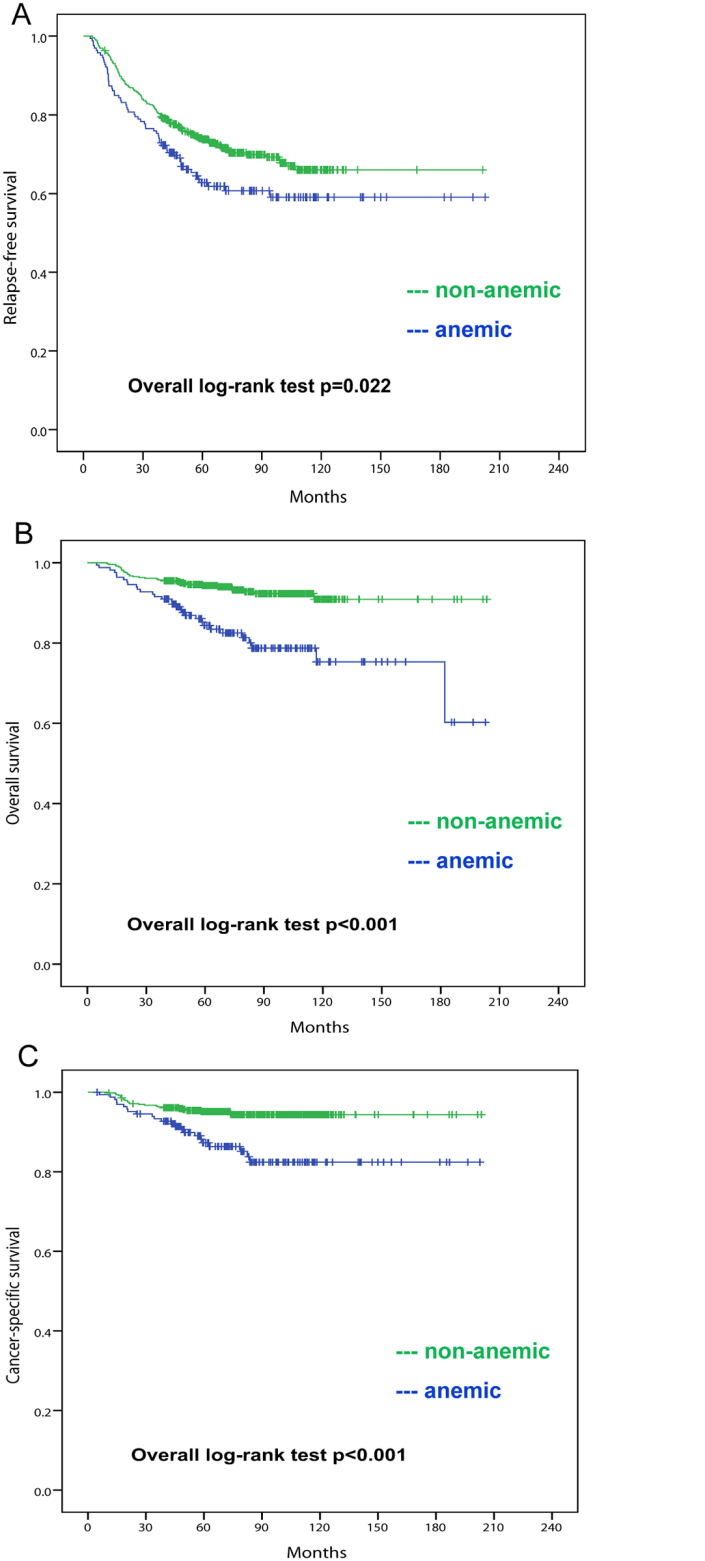
Relapse-free survival (A), overall survival (B) and cancer-specific survival (C) for patients receiving neoadjuvant chemotherapy with (blue) or without (green) pretreatment anemia. Unadjusted comparison of estimates was made by log-rank test.

By the last follow-up on December 31, 2014, 203 (31.0%) relapse events and 64 (9.8%) deaths have occurred. The median duration of follow-up was 76 months. As shown in Table 2, patients in the anemic group displayed significantly higher rates of recurrence (37.3% vs 28.8%, p = 0.042) and mortality (18.7% vs 6.7%, p<0.001) compared with their non-anemic counterparts. 10-year RFS was 59.1% for the anemic group and 66.0% for the non-anemic group respectively (Fig 1A, p = 0.022 by log-rank test). Additionally, the anemic group presented inferior 10-year OS (Fig 1B, 75.3% vs 90.9%, p<0.001) and CSS (Fig 1C, 82.4% vs 94.4%, p<0.001) when contrasted with the non-anemic group.

In order to further explore factors affecting the link between anemia and disease outcomes, we carried out subgroup analysis based on history of adjuvant radiotherapy (Table 3). 76.2% of patients (n = 499) were given radiotherapy following surgery and among these individuals baseline anemia was correlated with notably higher rates of relapse and death. Intriguingly enough, for radiation-naive patients (n = 156) preexisting anemia did not exhibit any differentiating role in disease-specific prognosis, although we did observe an overall survival disadvantage in patients with pretreatment anemia (p = 0.041).

**Table 3:** Correlation of pretreatment anemia with outcomes of patients with or without adjuvant radiotherapy.

N (%)	Adjuvant radiotherapy N = 499				No adjuvant radiotherapy N = 156			
	Hb<12g/dL	Hb≥12g/dL			Hb<12g/dL	Hb≥12g/dL		
	N = 124 (24.8%)	N = 375 (75.2%)	χ^2^	p	N = 42 (26.9%)	N = 114 (73.1%)	χ^2^	p
Relapse								
No	72(58.1)	274(73.1)	9.865	0.002	32(76.2)	74(64.9)	1.793	0.181
Yes	52(41.9)	101(26.9)			10(23.8)	40(35.1)		
Cancer-specific death								
No	105(84.7)	357(95.2)	15.030	<0.001	38(90.5)	107(93.9)	0.536	0.464
Yes	19(15.3)	18(4.8)			4(9.5)	7(6.1)		
Death								
No	100(80.6)	349(93.1)	15.947	<0.001	35(83.3)	107(93.9)	4.163	0.041
Yes	24(19.4)	26(6.9)			7(16.7)	7(6.1)		

Table 4 exhibits the prognostic value of candidate factors by multivariate Cox regression analysis. Pretreatment anemia was associated with elevated risk of relapse (hazard ratio (HR) 1.453, 95% CI 1.077–1.962, p = 0.015). Furthermore, patients with baseline anemia were more likely to die from breast cancer compared with non-anemic women (HR 2.961, 95% CI 1.679–5.222, p<0.001). Also, after ruling out the influence of confounding elements, pretreatment anemia was demonstrated to be independently predictive of higher all-cause mortality (HR 2.873, 95% CI 1.757–4.699, p<0.001). When further assessed as a continuous variable, pretreatment Hb level was again demonstrated to be a prognostic factor, with lower Hb concentrations related to inferior CSS (HR 0.979, 95% CI 0.961–0.997, p = 0.021) and OS (HR 0.977, 95% CI 0.962–0.992, p = 0.003).

**Table 4:** Multivariate analysis of prognostic factors for RFS, CSS and OS.

Variables	RFS		CSS		OS	
	HR (95% CI)	p	HR (95% CI)	p	HR (95% CI)	p
BMI	1.373(1.038–1.816)	0.026	NS	NS	NS	NS
Histology	NS	NS	2.304(1.115–4.762)	0.024	1.979(1.029–3.806)	0.041
Estrogen receptor status	NS	NS	3.681(2.025–6.694)	<0.001	NS	NS
Clinical node status	1.823(1.170–2.838)	0.008	NS	NS	NS	NS
Pathologic node status	5.255(3.284–8.410)	<0.001	5.882(2.311–14.969)	<0.001	2.840(1.444–5.586)	0.002
Pathologic stage	1.386(0.538–3.568)	0.499	NS	NS	NS	NS
Lymphovascular invasion	1.977(1.427–2.738)	<0.001	NS	NS	NS	NS
Adjuvant radiotherapy	1.877(1.341–2.629)	<0.001	NS	NS	NS	NS
Endocrine therapy	2.093(1.565–2.799)	<0.001	NS	NS	NS	NS
Anemia	1.453(1.077–1.962)	0.015	2.961(1.679–5.222)	<0.001	2.873(1.757–4.699)	<0.001
Baseline Hb level	0.993(0.984–1.002)	0.122	0.979(0.961–0.997)	0.021	0.977(0.962–0.992)	0.003

Abbreviations: RFS: relapse-free survival; CSS: cancer-specific survival; OS: overall survival; HR: hazard ratio; CI: confidence interval; Ref: reference group; NS: non-statistically significant.

## Discussion

In the present population-based study which reviewed a relatively large number of patients with breast cancer receiving NCT, we evaluated the predictive value of pretreatment anemia in outcomes of NCT. It was found that women complicated with anemia prior to NCT were less likely to achieve pCR compared with those non-anemic patients. Pretreatment anemia was associated with inferior pathological response to NCT as well as long-term prognosis in breast cancer.

As a common complication in patients with malignancy, anemia could possibly result from multiple etiological elements including predisposing factors, disease itself and treatment [15]. In our study which focused on anemia present before treatment, anemia was apparently not related to anti-cancer therapy. Predisposing iron deficiency due to menstrual cycles might contribute to low Hb levels prior to NCT [12]. As described by Table 1, there were a higher proportion of premenopausal women in anemic group (66.9%) than the non-anemic group (58.5%), which suggested a potential relationship between menopausal status and pretreatment anemia although this association failed to reach statistical significance. In addition, cancer per se could be a cause of pretreatment anemia. The ways in which cancer induces or aggravates anemia have been summarized [16]. Foremost was by generation of specific cytokines leading to functional iron deficiency, which then reduces production and longevity of red cells [17]. Also, cancer cells may directly infiltrate into bone marrow and suppress hematopoietic activity. Chronic hemorrhage at the tumor site caused by invasion of cancer cells might worsen anemia. Moreover, cancer-related anemia could be ascribed to inadequate intake of iron, folate and vitamin B12 secondary to anorexia. In this study, the effect of cancer on baseline Hb status could not be fully ruled out given that nearly 60% of reviewed cases were locally advanced. Patients in the anemic group tended to develop more advanced diseases (63.9% in stage III) compared with those with normal Hb levels (57.3% in stage III, p = 0.145). Similar finding was reported by Zhang et al that preoperative anemia was more frequent in breast cancer patients with higher clinical stage (p<0.001) [4].

The current population-based study provided clinical evidence with regard to the association between pretreatment anemia and poor response to NCT in breast cancer. In order to determine if baseline anemia was independently predictive of treatment efficacy, we performed detailed evaluation of critical confounders concerning preoperative chemotherapy. It was found that patients in both groups were given equal cycles of NCT with similar patterns of dosing reduction or delay during treatment. Given that the distribution of clinical and therapy-related elements was well balanced between groups, it would be relatively safe to conclude that pretreatment anemia was a predictor of response to NCT. One putative mechanism that underpinned the link between anemia and resistance to chemotherapy was tumor hypoxia. Particularly in breast cancer, hypoxia-related cellular resistance to anthracylines and taxanes, which were most widely used therapeutic drugs in clinical management of breast cancer, has been repeatedly investigated in vitro and in vivo. For anthracyclines, it was reported that under hypoxic condition P450 reductase interacted with anthracycline, which was then turned into a compound without anti-cancer activity following cleavage of a glycosidic bond [18]. Moreover, suppressed generation of reactive oxygen species, which participated in oxidative stress and cellular apoptosis, in cancer cells exposed to hypoxia represented another mechanism of resistance to anthracyclines [19]. Further studies revealed that hypoxia-inducible factor-1 (HIF-1) was essential in cellular adaptation to hypoxia and small interfering RNA targeting HIF-1 effectively overcame hypoxia-induced resistance to anthracyclines in breast cancer [20]. As for taxanes, AP-1, another crucial transcription factor, was also implicated in hypoxia-mediated protection against paclitaxel-induced apoptosis apart from HIF-1 [21]. Recent profiling study using whole transcriptome analysis identified gene TMEM45A to be involved in hypoxia-induced resistance to taxol in breast cancer cells [22].

Our study added to evidence with respect to the prognostic value of pretreatment anemia in patients with breast cancer. We demonstrated that anemia before treatment was predictive of worse response to NCT, which partly accounted for survival disadvantage observed in this subgroup. In addition to chemotherapy, radiotherapy was an indispensable modality in postoperative treatment of breast cancer and substantially improved local control of the disease. Response to ionizing irradiation was dependent on oxygenation status of tumor and studies have established tumor hypoxia as a predictor of unfavorable outcomes after radiotherapy in breast cancer [23,24]. Clinical findings about how Hb levels influence response to radiotherapy in breast cancer have been limited. In a prospective cohort study which involved women with single local recurrence lesion after mastectomy, the patients received radiotherapy following radical resection of recurrence lesion. Low Hb concentrations at the time of first relapse were found to correlate with shortened interval to second local failure or distant metastasis [25]. Another retrospective study reported that preoperative Hb concentration negatively affected DFS of patients receiving breast-conserving surgery followed by radiation rather than those undergoing mastectomy without radiotherapy [10]. Consistent with previous data, we found in subgroup analysis that the differentiating effects of pretreatment anemia on relapse and survival were manifest only in the patients who underwent radiotherapy after surgery, indicating that worse outcome associated with pretreatment anemia was also attributable to resistance to radiotherapy. Despite all this evidence, it is less likely that microscopic residual disease deposits are severely hypoxic as they can still obtain oxygen via passive diffusion. This raises the possibility that any residual disease arising from a background of preexisting anemia is more radioresistant and carries an inferior prognosis. Accordingly, the observed correlation between anemia and cancer prognosis should be independent of radiation history and our unplanned subgroup analysis might not be powered to reveal the anemia-related disparity in survival due to the small number of events in the radiation-free group. More large-scale studies are needed to elucidate the interaction between anemia, radiation and disease outcome in breast cancer.

At least two studies were carried out to evaluate the impact of baseline Hb levels on response to chemotherapy in neoadjuvant setting in breast cancer. In a cohort study enrolling 157 women, patients with baseline Hb≤13g/dL were less clinically responsive to NCT [13]. Yet another prospective study (n = 139) failed to reveal significant association between pretreatment Hb levels and response to NCT [14]. In the present study we assessed pretreatment Hb concentration as both categorical and continuous variable. As a categorical variable, pretreatment Hb level was demonstrated to be prognostic and predictive of response to NCT, while as a continuous variable it was prognostic but not predictive for NCT benefit. Compared with previous work, our current study was of greater clinical importance in the following aspects. To start with, a relatively large cohort of patients was subject to analysis which helped increase the power of this study and reliability of derived results. Second, different from earlier studies, we adopted pCR instead of clinical response as primary outcome of NCT given the widely accepted notion that pCR after NCT was a robust prognostic factor in breast cancer independent of other covariates. Factors considerably influencing rate of pCR were more clinically critical and worthy of further research. Moreover, previous findings were mostly obtained from studies assessing traditional chemotherapy regimens such as CMF, which could not be equally applied to current clinical practice. Our study nonetheless, evaluated effect of anemia among patients treated with regimens containing both anthracyclines and taxanes, which were most frequently administered agents in management of breast cancer nowadays. Last but not least, our data initially added to evidence for the impact of baseline anemia on NCT outcomes among Chinese women with breast cancer. Further exploration is needed to characterize the racial disparity in cancer-related anemia and its prognostic value.

Residual tumors after NCT were in essence composed by chemo-resistant subclones which contributed to cancer progression and distant metastasis. Considering the relatively low response to chemotherapy and survival disadvantage ascribed to pretreatment anemia in breast cancer, physicians are justified to carefully evaluate the condition and provide sound interventions for anemic patients especially in neoadjuvant setting. For now three treatment options are available for correction of cancer-related anemia including iron supplement, packed red cell transfusion and erythropoiesis-stimulating agents (ESA). Iron supplement, either intravenous or oral, was indicated only in those with definite iron deficiency confirmed by relevant blood biochemical tests. Our study suggested that menstruation-caused iron deficiency could partly account for pretreatment anemia, which rationalized routine assessment of nutritional status in premenopausal cancer patients with anemia. What is more, in the present analysis a vast majority of anemic patients with anemia (92.2%) was mildly anemic, for whom packed red cell transfusion was usually unnecessary. With regard to ESA, disappointing results have been released from a series of prospective clinical trials. Recently in a meta-analysis of randomized trials evaluating ESA use in patients with breast cancer, it was found that ESA use was associated with elevated risk of disease progression and mortality [26]. Pre-clinical data indicated ESA protected breast cancer stem-like cells from cytotoxic agents in vitro [27] and promoted metastasis in vivo [28]. The aforementioned evidence has led to restricted use of ESA, which was recommended only in combination with chemotherapy in metastatic breast cancer patients with Hb<10g/dL [29]. Data from large cohort studies also highlighted the link between blood transfusion and increased risk of thrombotic events as well as mortality in cancer patients [30]. Yet caution should be taken when we interpreted the paradoxical influence of anemia correction on disease outcomes. Treatment-related adverse events such as thromboembolism and potential tumor-promoting mechanisms probably accounted for the negative prognostic effect of options including ESA and red cell transfusion, which provided rationale for exploration of better alternatives with fewer side effects. On the other hand, as previously discussed tumors developing in the setting of preexisting anemia were likely to be more stem-like with greater potential of invasion and metastasis, so correction of anemia after diagnosis of malignancy might be ineffective and even counterproductive. In future, molecular therapeutics targeting aberrant signaling pathways implicated in hypoxia-induced resistance may present as promising strategy. Further prospective randomized studies are warranted to identify optimal interventions and improve the prognosis of this subgroup.

## Conclusions

The present population-based study demonstrated that pretreatment anemia was related to worse pathological response to NCT as well as survival status in breast cancer. Further studies are warranted to identify optimal interventions and improve the prognosis of this subgroup.

## References

[1] Knight K, Wade S, Balducci L. Prevalence and outcomes of anemia in cancer: a systematic review of the literature. Am J Med. 2004; 116 Suppl 7A:11–26. 10.1016/j.amjmed.2003.12.008 15050883

[2] Caro JJ, Salas M, Ward A, Goss G. Anemia as an independent prognostic factor for survival in patients with cancer: a systemic, quantitative review. Cancer. 2001; 91:2214–2221. 11413508

[3] Boehm DU, Lebrecht A, Schmidt M, Siggelkow W, Lindner C, Litz A, et al Prognostic impact of haemoglobin levels in breast cancer. Anticancer Res. 2007; 27:1223–1226. 17465267

[4] Zhang Y, Chen Y, Chen D, Jiang Y, Huang W, Ouyang H, et al Impact of preoperative anemia on relapse and survival in breast cancer patients. BMC Cancer. 2014; 14:844 10.1186/1471-2407-14-844 25406979 PMC4242544

[5] Vaupel P, Thews O, Hoeckel M. Treatment resistance of solid tumors: role of hypoxia and anemia. Med Oncol. 2001; 18:243–259. 11918451 10.1385/MO:18:4:243

[6] Harrison LB, Chadha M, Hill RJ, Hu K, Shasha D. Impact of tumor hypoxia and anemia on radiation therapy outcomes. Oncologist. 2002; 7:492–508. 12490737 10.1634/theoncologist.7-6-492

[7] Hu K, Harrison LB. Impact of anemia in patients with head and neck cancer treated with radiation therapy. Curr Treat Options Oncol. 2005; 6:31–45. 15610713 10.1007/s11864-005-0011-4

[8] Grogan M, Thomas GM, Melamed I, Wong FL, Pearcey RG, Joseph PK, et al The importance of hemoglobin levels during radiotherapy for carcinoma of the cervix. Cancer. 1999; 86:1528–1536. 10526282 10.1002/(sici)1097-0142(19991015)86:8<1528::aid-cncr20>3.0.co;2-e

[9] Dunphy EP, Petersen IA, Cox RS, Bagshaw MA. The influence of initial hemoglobin and blood pressure levels on results of radiation therapy for carcinoma of the prostate. Int J Radiat Oncol Biol Phys. 1989; 16:1173–1178. 2715066 10.1016/0360-3016(89)90277-0

[10] Henke M, Sindlinger F, Ikenberg H, Gerds T, Schumacher M. Blood hemoglobin level and treatment outcome of early breast cancer. Strahlenther Onkol. 2004; 180:45–51. 14704844 10.1007/s00066-004-1123-7

[11] Green SL, Giaccia AJ. Tumor hypoxia and the cell cycle: implications for malignant progression and response to therapy. Cancer J Sci Am. 1998; 4:218–223. 9689978

[12] Dubsky P, Sevelda P, Jakesz R, Hausmaninger H, Samonigg H, Seifert M, et al Anemia is a significant prognostic factor in local relapse-free survival of premenopausal primary breast cancer patients receiving adjuvant cyclophosphamide/methotrexate/5-fluorouracil chemotherapy. Clin Cancer Res. 2008; 14:2082–2087. 10.1158/1078-0432.CCR-07-2068 18381948

[13] Bottini A, Berruti A, Brizzi MP, Bersiga A, Generali D, Allevi G, et al Pretreatment haemoglobin levels significantly predict the tumour response to primary chemotherapy in human breast cancer. Br J Cancer. 2003; 89:977–982. 12966412 10.1038/sj.bjc.6601216 PMC2376950

[14] Beresford MJ, Burcombe R, Ah-See ML, Stott D, Makris A. Pre-treatment haemoglobin levels and the prediction of response to neoadjuvant chemotherapy in breast cancer. Clin Oncol (R Coll Radiol). 2006; 18:453–458. 16909968 10.1016/j.clon.2006.04.006

[15] Gilreath JA, Stenehjem DD, Rodgers GM. Diagnosis and treatment of cancer-related anemia. Am J Hematol. 2014; 89:203–212. 10.1002/ajh.23628 24532336

[16] Wilson J, Yao GL, Raftery J, Bohlius J, Brunskill S, Sandercock J, et al A systematic review and economic evaluation of epoetin alpha, epoetin beta and darbepoetin alpha in anaemia associated with cancer, especially that attributable to cancer treatment. Health Technol Assess. 2007; 11:1–202. 10.3310/hta11130 17408534

[17] Thompson CA, Steensma DP. Pure red cell aplasia associated with thymoma: clinical insights from a 50-year single-institution experience. Br J Haematol. 2006; 135:405–407. 17032177 10.1111/j.1365-2141.2006.06295.x

[18] Pan SS, Pedersen L, Bachur NR. Comparative flavoprotein catalysis of anthracycline antibiotic. Reductive cleavage and oxygen consumption. Mol Pharmacol. 1981; 19:184–186. 6937729

[19] Matthews NE, Adams MA, Maxwell LR, Gofton TE, Graham CH. Nitric oxide-mediated regulation of chemosensitivity in cancer cells. J Natl Cancer Inst. 2001; 93:1879–1885. 11752013 10.1093/jnci/93.24.1879

[20] Sullivan R, Pare GC, Frederiksen LJ, Semenza GL, Graham CH. Hypoxia-induced resistance to anticancer drugs is associated with decreased senescence and requires hypoxia-inducible factor-1 activity. Mol Cancer Ther. 2008; 7:1961–1973. 10.1158/1535-7163.MCT-08-0198 18645006

[21] Flamant L, Notte A, Ninane N, Raes M, Michiels C. Anti-apoptotic role of HIF-1 and AP-1 in paclitaxel exposed breast cancer cells under hypoxia. Mol Cancer. 2010; 9:191 10.1186/1476-4598-9-191 20626868 PMC3098009

[22] Flamant L, Roegiers E, Pierre M, Hayez A, Sterpin C, De Backer O, et al TMEM45A is essential for hypoxia-induced chemoresistance in breast and liver cancer cells. BMC Cancer. 2012; 12:391 10.1186/1471-2407-12-391 22954140 PMC3519606

[23] Vaupel P, Mayer A. Hypoxia and anemia: effects on tumor biology and treatment resistance. Transfus Clin Biol. 2005; 12:5–10. 15814285 10.1016/j.tracli.2004.11.005

[24] Feldmann HJ. Oxygenation of human tumors—implications for combined therapy. Lung Cancer. 2001; 33 Suppl 1:77–83. 10.1016/s0169-5002(01)00306-3 11576711

[25] Kamby C, Sengelov L. Survival and pattern of failure following locoregional recurrence of breast cancer. Clin Oncol (R Coll Radiol). 1999; 11:156–163. 10465468 10.1053/clon.1999.9033

[26] Aapro M, Moebus V, Nitz U, O'Shaughnessy J, Pronzato P, Untch M, et al Safety and efficacy outcomes with erythropoiesis-stimulating agents in patients with breast cancer: a meta-analysis. Ann Oncol. 2014; 10.1093/annonc/mdu579 25542926

[27] Todaro M, Turdo A, Bartucci M, Iovino F, Dattilo R, Biffoni M, et al Erythropoietin activates cell survival pathways in breast cancer stem-like cells to protect them from chemotherapy. Cancer Res. 2013; 73:6393–6400. 10.1158/0008-5472.CAN-13-0248 24008319

[28] Hedley BD, Chu JE, Ormond DG, Beausoleil MS, Boasie A, Allan AL, et al Recombinant human erythropoietin in combination with chemotherapy increases breast cancer metastasis in preclinical mouse models. Clin Cancer Res. 2011; 17:6151–6162. 10.1158/1078-0432.CCR-10-3298 21856770

[29] Rodgers GM 3rd, Becker PS, Blinder M, Cella D, Chanan-Khan A, Cleeland C, et al Cancer- and chemotherapy-induced anemia. J Natl Compr Canc Netw. 2012; 10:628–653. 22570293 10.6004/jnccn.2012.0064

[30] Khorana AA, Francis CW, Blumberg N, Culakova E, Refaai MA, Lyman GH. Blood transfusions, thrombosis, and mortality in hospitalized patients with cancer. Arch Intern Med. 2008; 168: 2377–2381. 10.1001/archinte.168.21.2377 19029504 PMC2775132

